# Supplementary material for: Different Strategies to Attenuate the Toxic Effects of Zinc Oxide Nanoparticles on Spermatogonia Cells
Source: Nanomaterials (Basel). 2022 Oct 11;12(20):3561. doi: 10.3390/nano12203561 (PMC9607034; doi:10.3390/nano12203561)
Supplement: Supplementary file 1 [file nanomaterials-12-03561-s001.zip › nanomaterials-1917088-supplementary.pdf]

nanomaterials

MDPI

Supplementary Material

Different Strategies to Attenuate the Toxic Effects of Zinc Oxide Nanoparticles on Spermatogonia Cells

Mariana Vassal [1], Cátia D. Pereira [2], Filipa Martins [2], Vera L. M. Silva [3], Artur M. S. Silva [3], Ana M. R. Senos [4,5], Maria Elisabete V. Costa [4,5], Maria de Lourdes Pereira [4,6,*,†] and Sandra Rebelo [2,*,†]

1 Department of Biology, University of Aveiro, 3810-193 Aveiro, Portugal
2 Institute of Biomedicine—iBiMED, Department of Medical Sciences, University of Aveiro, 3810-193 Aveiro, Portugal
3 LAQV-REQUIMTE, Department of Chemistry, University of Aveiro, 3810-193 Aveiro, Portugal
4 CICECO-Aveiro Institute of Materials, University of Aveiro, 3810-193 Aveiro, Portugal
5 Department of Materials and Ceramic Engineering, University of Aveiro, 3810-193 Aveiro, Portugal
6 Department of Medical Sciences, University of Aveiro, 3810-193 Aveiro, Portugal
* Correspondence: mlourdespereira@ua.pt (M.d.L.P.); srebelo@ua.pt (S.R.)
† These authors contributed equally to this work.

Citation: Vassal, M.; Pereira, C.D.; Martins, F.; Silva, V.L.M.; Silva, A.M.S.; Senos, A.M.R.; Costa, M.E. V. ; de Lourdes Pereira, M.; Rebelo, S. Different Strategies to Attenuate the Toxic Effects of Zinc Oxide Nanoparticles on Spermatogonia Cells. *Nanomaterials* **2022**, *12*, 3561. https://doi.org/10.3390/nano12203561

Academic Editors: Wenyan Yin and Rosalia Bertorelli

Received: 30 August 2022
Accepted: 7 October 2022
Published: 11 October 2022

Publisher's Note: MDPI stays neutral with regard to jurisdictional claims in published maps and institutional affiliations.

Copyright: © 2022 by the authors. Licensee MDPI, Basel, Switzerland. This article is an open access article distributed under the terms and conditions of the Creative Commons Attribution (CC BY) license (https://creativecommons.org/licenses/by/4.0/).

1. Supplementary Data

*1.1. Synthesis and structural characterization of (E)-3-(2,6-dichlorophenyl)-1-(2-hydroxyphenyl)prop-2-en-1-one (**1**)*

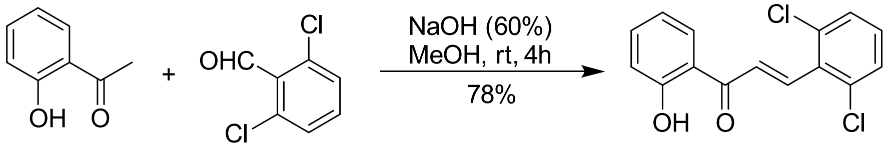

1

Experimental procedure: An aqueous solution of sodium hydroxide (60%, 160 mL) was slowly added to a methanolic solution (150 mL) of 2′-hydroxyacetophenone (4 mL, 33 mmol). After cooling the solution to room temperature, the 2,6-dichlorobenzaldehyde (7.0 g, 40 mmol) was added. The reaction mixture was stirred at room temperature for 4 h. After this period, it was poured over a mixture of ice and water, and an aqueous hydrochloric acid solution was added until pH 2. The precipitate formed was filtered, dissolved in chloroform (300 mL), and washed with a 5% aqueous solution of sodium hydrogen carbonate (2 x 200 mL). The organic layer was collected, dried over anhydrous sodium sulfate and the solvent was evaporated to dryness. The obtained residue was crystallized from ethanol. After filtration, the (*E*)-3-(2,6-dichlorophenyl)-1-(2-hydroxyphenyl)prop-2-en-1-one (**1**) was isolated as a pure yellow solid.

(*E*)-3-(2,6-dichlorophenyl)-1-(2-hydroxyphenyl)prop-2-en-1-one (**1**), yellow solid, 7.54 g, 78% yield (mp 101.8–102.5 °C).

^{1}H NMR (300.13 MHz, $CDCl_3$) δ = 6.95 (ddd, *J* = 8.2, 7.1, 1.2 Hz, 1H, H-5′), 7.04 (dd, *J* = 8.5, 1.2 Hz, 1H, H-3′), 7.23 (t, *J* = 8.5 Hz, 1H, H-4), 7.41 (d, *J* = 8.5 Hz, 2H, H-3,5), 7.52 (ddd, *J* = 8.5, 7.1, 1.6 Hz, 1H, H-4′), 7.84 (d, *J* = 16.0 Hz, 1H, H-α), 7.85 (dd, *J* = 8.2, 1.6 Hz, 1H, H-6′), 7.99 (d, *J* = 16.0 Hz, 1H, H-β), 12.63 (s, 1H, 2′-O<u>H</u>) ppm.

^{13}C NMR (75.47 MHz, $CDCl_3$) δ = 118.7 (C-3′), 119.1 (C-5′), 119.9 (C-1′), 128.8 (C-α), 129.0 (C-3,5), 130.0 (C-6′), 130.2 (C-4), 132.3 (C-1), 135.4 (C-2,6), 136.8 (C-4′), 138.4 (C-β), 163.7 (C-2′), 193.7 (C=O) ppm.

2. Supplementary Figures

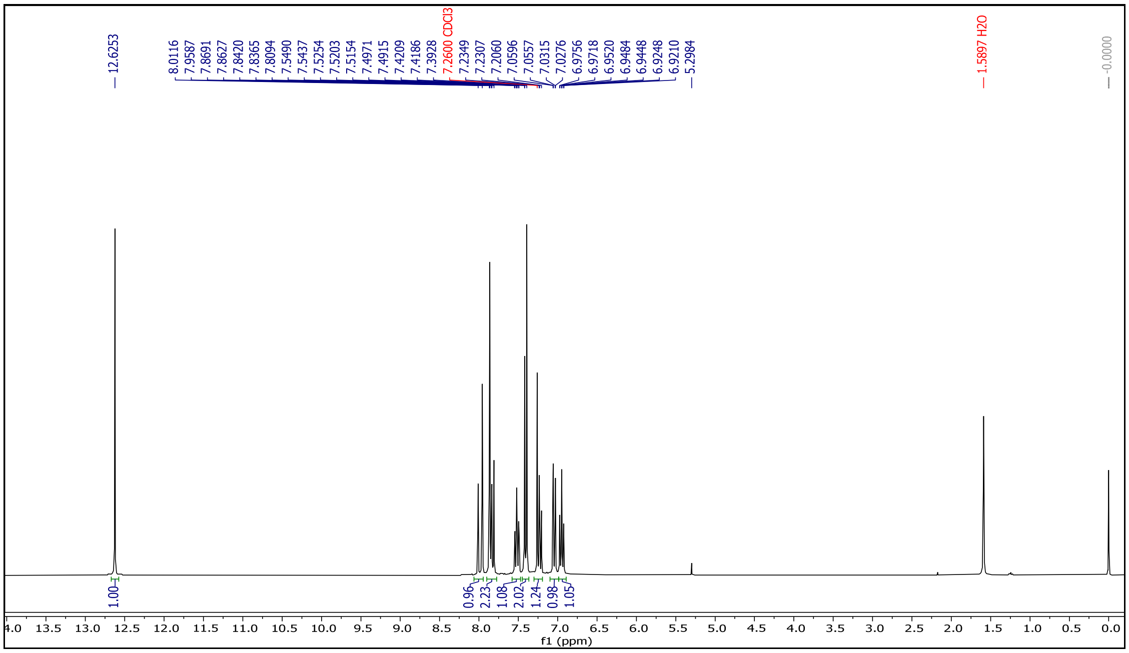

Figure S1. ^{1}H NMR spectrum of (*E*)-3-(2,6-dichlorophenyl)-1-(2-hydroxyphenyl)prop-2-en-1-one (**1**) (300.13 MHz, $CDCl_3$).

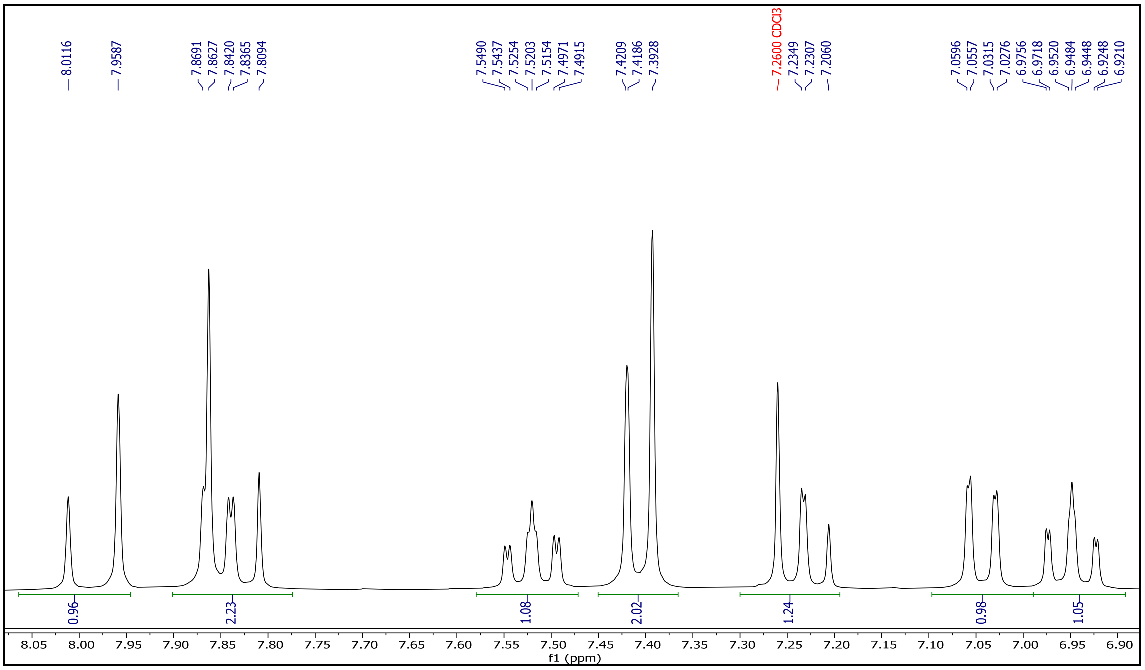

Figure S2. Expansion of the ^{1}H NMR spectrum of (*E*)-3-(2,6-dichlorophenyl)-1-(2-hydroxyphenyl)prop-2-en-1-one (**1**) (300.13 MHz, $CDCl_3$).

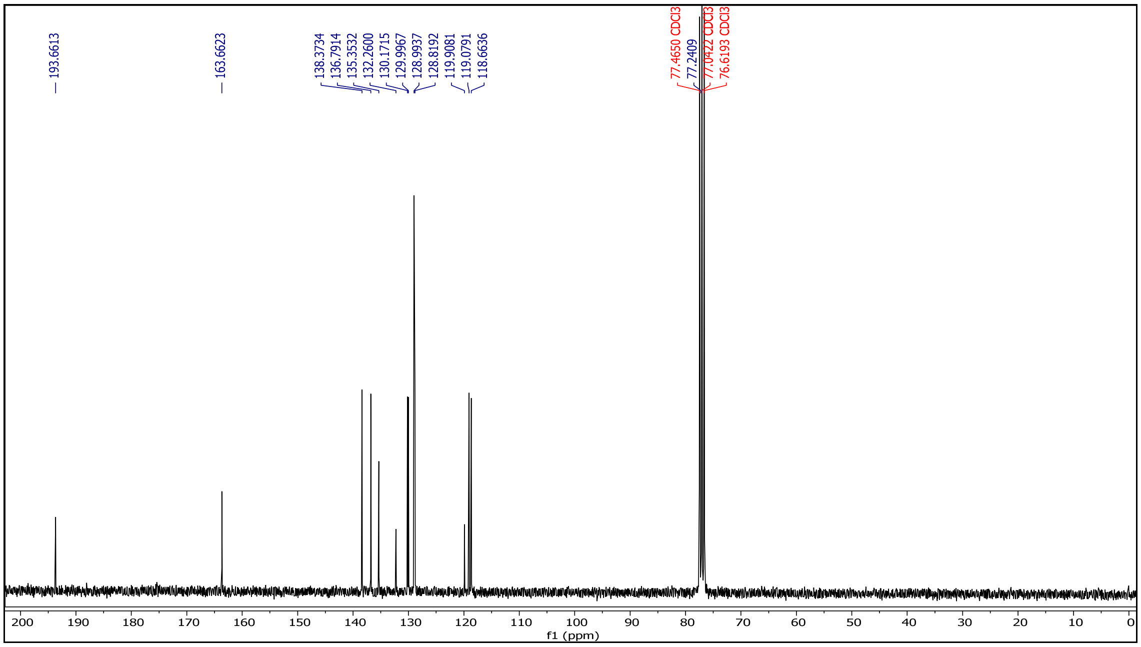

Figure S3. ^{13}C NMR spectrum of (*E*)-3-(2,6-dichlorophenyl)-1-(2-hydroxyphenyl)prop-2-en-1-one (**1**) (75.47 MHz, $CDCl_3$).

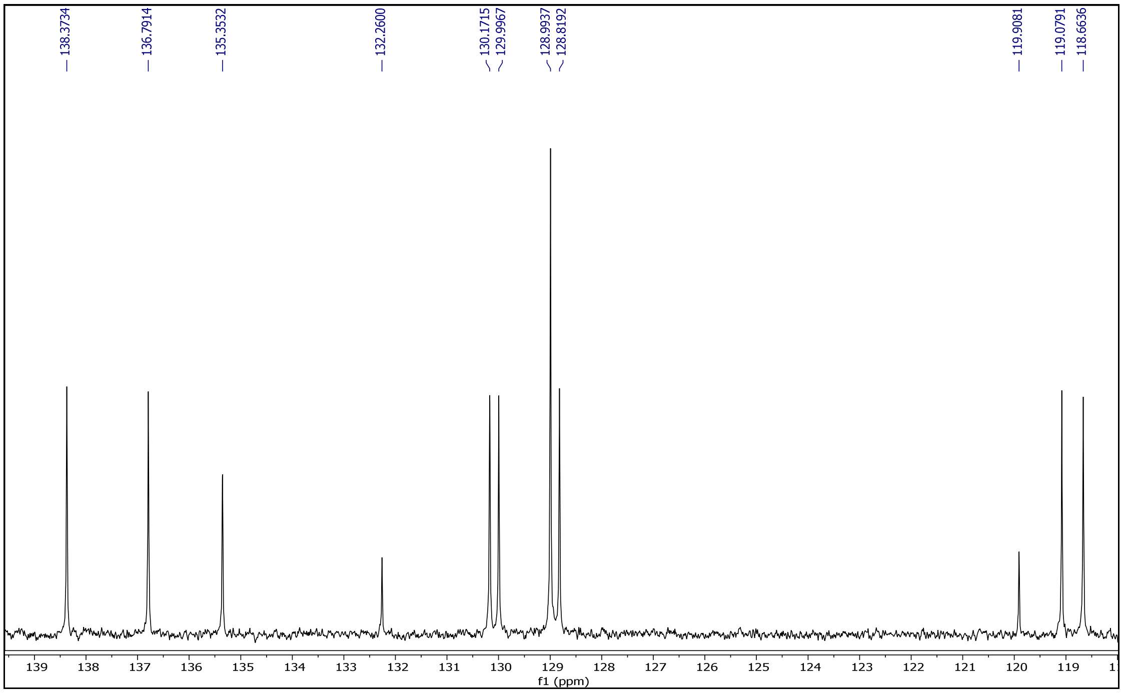

Figure S4. Expansion of the ^{13}C NMR spectrum of (*E*)-3-(2,6-dichlorophenyl)-1-(2-hydroxyphenyl)prop-2-en-1-one (**1**) (75.47 MHz, $CDCl_3$).

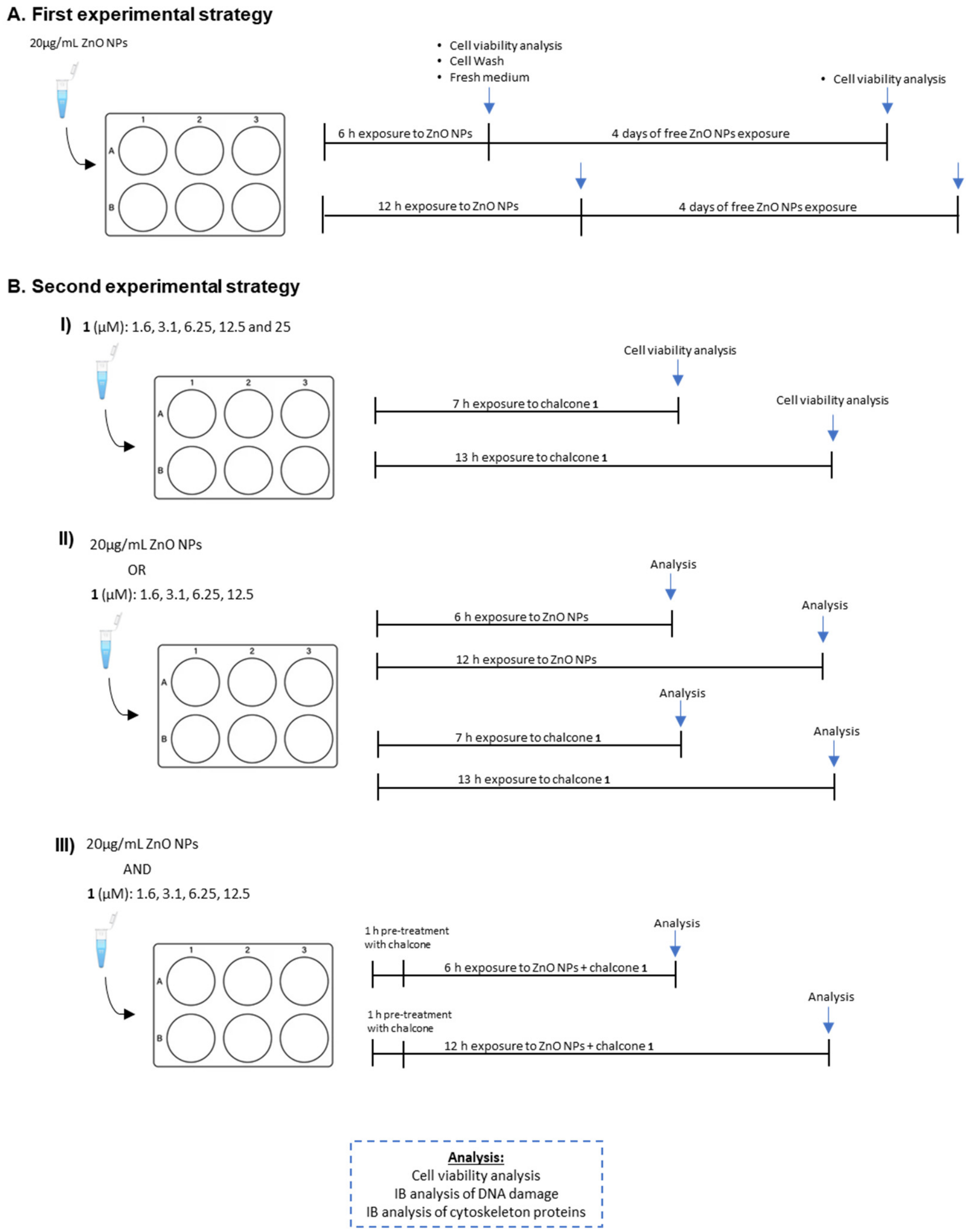

Figure S5. Schematic diagram of the experimental designs used. A - Schematic diagram of the first experimental design used. The exposure times for the experiments as well as the recovery period. Blue arrows indicate the timepoints where the cell viability was monitored. **B-** Schematic diagram of the second experimental design used. First, (I) cells were incubated only with different chalcone **1** concentrations (0-25 µM) for 7 and 13 h to test the cytotoxicity of the compound. Then, cells were exposed to chalcone **1** (0-12.5 µM) or 20 µg/mL of ZnO NPs (II) in the absence and presence of different concentration of chalcone **1** (0-12.5 µM). III) Cells were pretreated with chalcone **1** for 1 h and co-exposed to ZnO NPs and chalcone **1** to evaluate the protective effects of chalcone **1** against ZnO NPs toxicity. IB, immunoblotting; NPs, nanoparticles.

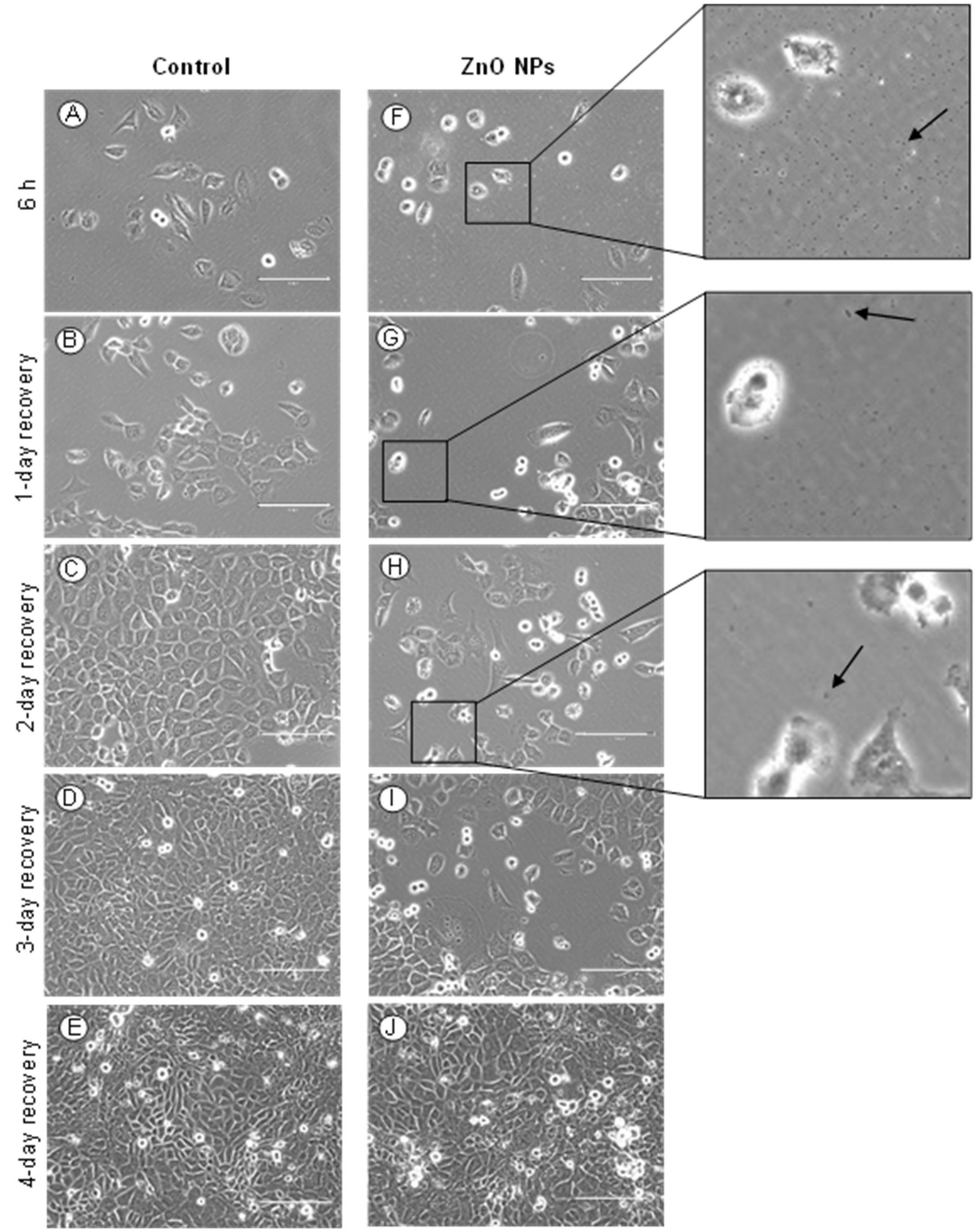

Figure S6. GC-1 cells monitoring by light microscopy (6 h). Light microscopy images of GC-1 cells at different time points; (A-E) unexposed cells, (F-J) cells exposed to 20 µg/mL of ZnO NPs for 6 h. Black arrows are pointing into visible aggregates of ZnO NPs. Photos were taken at 20x magnification. Bar corresponds to 150 µm.

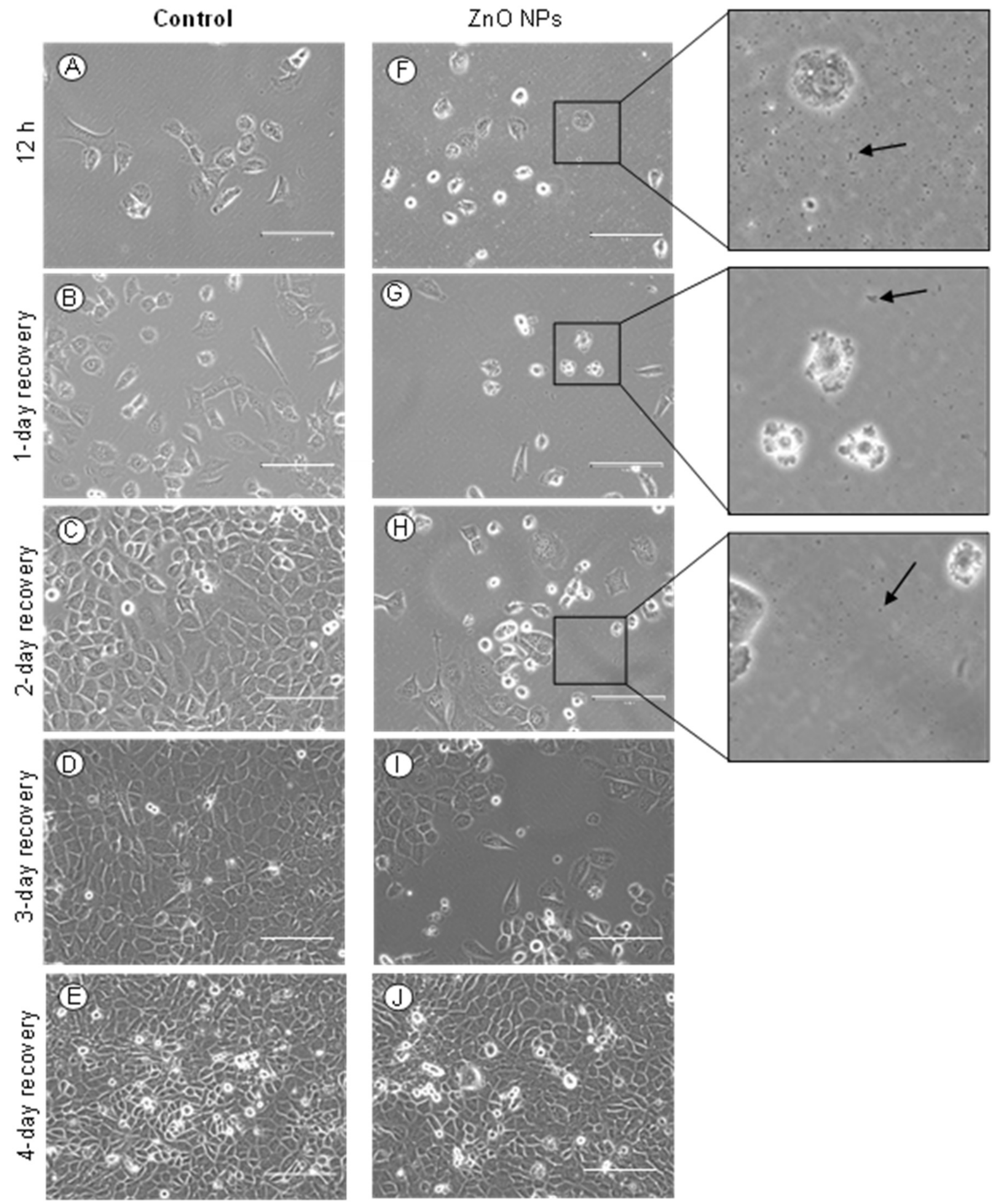

Figure S7. GC-1 cells monitoring by light microscopy (12 h). Light microscopy images of GC-1 cells at different time points; (A-E) unexposed cells, (F-J) cells exposed to 20 µg/mL of ZnO NPs for 12 h. Black arrows are pointing into visible aggregates of ZnO NPs. Photos were taken at 20x magnification. Bar corresponds to 150 µm.
